# Editorial: Development of the vestibular system

**DOI:** 10.3389/fneur.2023.1191086

**Published:** 2023-03-31

**Authors:** Mathieu Beraneck, Karen L. Elliott, Joel C. Glover, Hans Straka

**Affiliations:** ^1^CNRS UMR 8002, INCC - Integrative Neuroscience and Cognition Center, Université Paris Cité, Paris, France; ^2^Department of Biology, The University of Iowa, Iowa City, IA, United States; ^3^Department of Molecular Medicine, Institute of Basic Medical Sciences, University of Oslo, Oslo, Norway; ^4^Faculty of Biology, Ludwig-Maximilians-University, Munich, Germany

**Keywords:** otic placode, developmentally regulated genes, ontogeny, circuit formation, behavioral maturation

*The vestibular system is the dominating sensory element to detect head/body motion. The encoded signals are essential to stabilize gaze and posture during locomotion, to control autonomic function, and to update navigation circuits to orient and navigate in space. Thus, all animals require functional peripheral structures and pathways as well as central networks to ensure adequate sensory–motor transformations that allow efficient locomotor activity as soon as the respective motor elements have been developed. The diversity of endorgans and neuronal elements in the inner ear are formed through precise genetic instructions and timelines. These latter events create parallel and sequential elements that allow the encoding and transmitting of spatially and dynamically diverse sensory information during body motion in space. During this period, neural computations must be rapidly implemented to ensure the execution of adequate behavioral reactions that generate visual and postural stability. Accordingly, the developmental establishment of a functional vestibular system is key for goal-oriented locomotion*.

This text was proposed as an introduction by Professor Dr. Hans Straka, who initiated this Research Topic. As a neurophysiologist, Hans was passionate about the development of the vestibular system as a way to understand the molecular, cellular, microcircuit, and circuit organizations that allow the vestibular system to provide optimal sensory inputs for gaze stabilization, postural control, balance, and orientation in various contexts. His interest in complementary aspects of the development of the vestibular system is well illustrated by his list of publications, which, for instance, covers its genetic regulation in *Rhombomeric organization of the vestibular pathways* (1), the influence of the ontogeny of the inner ear organs in *Semicircular canal size determines the developmental onset of angular vestibulo-ocular reflexes in larval Xenopus* (2), the maturation of neuronal elements (3), the establishment of functional reflexes in *Ontogenetic rules and constraints of vestibulo-ocular reflex development* (4), and the acquisition of locomotor capacities in *Stabilization of Gaze during Early Xenopus Development by Swimming-Related Utricular Signals* (5). This multi-layered approach was central to Hans' research, as were the interspecies comparisons performed through eco-physiological and evolutionary perspectives [see, for instance, *Connecting ears to eye muscles: evolution of a “simple” reflex arc* (6)].

The papers published in this Research Topic correspond well to the basic vestibular research that Hans greatly appreciated. In their perspective paper, “*The Larval Zebrafish Vestibular System Is a Promising Model to Understand the Role of Myelin in Neural Circuits,”* Auer and Schoppik propose that vestibular reflex circuits are well suited to assess the functional consequences of myelination during development and demyelination during disease. In particular, they argue that the larval zebrafish vestibular system is a model of choice, because with it scientists can leverage the zebrafish's cutting-edge genetic and optical technologies with the careful and quantitative measurements of behavior, for example, during balance regulation. The advantages of this animal model to the understanding of vestibular system development are further demonstrated by the paper by Jia and Bagnall titled “*Monosynaptic Targets of Utricular Afferents in the Larval Zebrafish***”**. In this original research contribution, the authors report on anatomical data at a subcellular, cellular, and circuit level. The data were collected using the high-resolution serial-section electron microscopy of a larval zebrafish at an early developmental stage. The snapshot taken early in development appears helpful for evaluating the developmental sequence in the vestibular system, in particular based on the assumption that the earliest born neurons are likely to be myelinated first. This report is specifically focused on utricular afferents and their central projections in the brainstem, which are segregated based on their anatomical location and inferred utricular tuning. Utricular signals are functionally essential, and they are the sole source of vestibular information about head movement and orientation at these early larval stages.

Genetic regulation of the development of the vestibular system is addressed by the original research article provided by Stoner et al. titled “*Fzd3 Expression Within Inner Ear Afferent neurons Is Necessary for Central Pathfinding*”. Taking advantage of genetically engineered mouse strains combined with a conditional knockout strategy, they elegantly demonstrate that the expression of Fzd3 specifically in the vestibular afferents, and not in the hair cells, regulates the central pathfinding within the hindbrain. This paper on the peripheral development of the vestibular system in mice is complemented by another original research article concerned with the maturation of central vestibular neurons. Dubois et al., in their contribution titled “*Perinatal Development of Central Vestibular Neurons in Mice*”, combine a transgenic mouse model and retrograde labeling to compare the electrophysiological maturation of inhibitory neurons implicated in the commissural and lateral vestibulospinal pathways, respectively. The acquisition of specific functional identities from the embryonic to postnatal stages varied between the two pathways, with more maturation observed in the LVST neurons during the perinatal period. The changes reported parallel the maturation of the postural reflexes and likely also the development of vestibular afferent activity.

The Research Topic also included one case report by Liu et al. titled “*Vestibular paroxysmia associated with congenital vascular malformation”*, which emphasizes that beyond the fundamental research conducted on animal models, studying the mechanisms that govern the ontogeny of the vestibular system is necessary to understand the congenital pathologies encountered in human patients.

## Dedication

Prof. Dr. Hans Straka (1961–2022) was awarded the Hallpike Nylen medal in 2022 by the international Bárány Society for his outstanding contributions to basic vestibular science. We dedicate this Research Topic to the memory of our dearly missed colleague and friend.

## Author contributions

MB wrote the first draft of the manuscript, based on the original text provided by HS. MB, KE, and JG read, revised and approved the submitted version.
